# Hippo signaling pathway in polycystic ovary syndrome

**DOI:** 10.3389/fendo.2025.1623143

**Published:** 2025-10-06

**Authors:** Jiahui Wen, Guan Cheng, Yan Zhang, Su Liu

**Affiliations:** ^1^ Department of Clinical Laboratory, Institute of Translational Medicine, Renmin Hospital of Wuhan University, Wuhan, Hubei, China; ^2^ Shenzhen Key Laboratory for Reproductive Immunology of Peri-implantation, Shenzhen Zhongshan Institute for Reproductive Medicine and Genetics, Shenzhen Zhongshan Obstetrics and Gynecology Hospital (Formerly Shenzhen Zhongshan Urology Hospital), Shenzhen, Guangdong, China

**Keywords:** Hippo signaling pathway, polycystic ovary syndrome, YAP, TAZ, ovary, lipid metabolism, insulin resistance, inflammation

## Abstract

Polycystic ovary syndrome (PCOS) is a complex endocrine-metabolic disorder syndrome, that predominantly affects women of reproductive age. It is characterized by marked clinical heterogeneity involving multiple systems including reproductive, metabolic and immune systems, while existing diagnostic protocols remain inadequate for clinical needs. Moreover, the incomplete understanding of PCOS etiology has limited therapeutic strategies for symptomatic management rather than interventions targeting core pathological mechanisms, resulting in PCOS frequently persisting as a chronic condition with an increased risk of long-term complications such as type 2 diabetes, metabolic disorder-associated fatty liver disease and cardiovascular disease. This clinical reality underscores the urgent need to elucidate its pathogenic network at the molecular level. Emerging evidence suggests that the Hippo signaling pathway plays a central role in the pathological process of PCOS through dynamically regulating cell proliferation-apoptosis balance, differentiation programs and metabolic homeostasis. This review examines the molecular mechanisms governing Hippo signaling transduction and its physiological relevance, with a focused analysis of its diverse implications in PCOS pathophysiology, particularly in reproductive dysfunction, metabolic-endocrine disturbances, and immune dysregulation. These mechanistic insights not only advance our understanding of PCOS pathogenesis but also provide a theoretical foundation for developing signaling pathway-targeted precision therapies.

## 1 Introduction

PCOS, also known as Stein-Leventhal syndrome, is a complex endocrine-metabolic disorder affecting approximately 11-13% of reproductive-aged women globally (1). This condition is clinically characterized by impaired fertility, metabolic disorders and immune microenvironment dysregulation. Notably, current therapeutic interventions remain challenging, with patients facing significant risks of comorbidities such as type 2 diabetes mellitus, cardiovascular disease and non-alcoholic fatty liver disease (2). Furthermore, as a multisystem disorder involving reproductive, metabolic and immune interactions, systematic elucidation of PCOS pathogenesis holds critical implications for advancing clinical diagnosis and treatment strategies.

The Hippo signaling pathway, an evolutionarily conserved regulatory network, derives its name from the tissue hyperproliferation phenotype observed in *Drosophila melanogaster* with Hippo kinase mutations (3, 4). This pathway governs several biological processes including cell proliferation, apoptosis, differentiation and tissue homeostasis through phosphorylation cascades (5). Remarkably, the Hippo signaling pathway has multidimensional regulatory functions. In the female reproductive system, it regulates follicular developmental homeostasis, while its functional dysregulation is strongly associated with reproductive-endocrine disorders, such as PCOS and premature ovarian insufficiency (6–9). In addition, this pathway can also coordinate systemic metabolism by interfering with metabolites and/or metabolic signaling, as well as modulate immune microenvironment homeostasis through its involvement in immune cell differentiation and inflammatory cytokine secretion (10, 11).

As early as 2012, Li et al. identified the Hippo signaling pathway core effector YAP1 as a susceptibility gene for PCOS through genome-wide association study (GWAS) analysis (12). Subsequent advancements in research methodologies have established that Hippo signaling dysregulation contributes to PCOS pathogenesis via aberrant androgen biosynthesis, granulosa cell cycle disruption, and impaired folliculogenesis (13). However, given the multisystem complexity of PCOS, the mechanistic studies need to break through the traditional single-system analysis framework and conduct comprehensive analysis from a holistic perspective. As evidenced by extensive literature reviews, current research on the Hippo-PCOS relationship remains limited. This review summarizes and discusses the roles of the Hippo signaling pathway and its key components in reproductive, metabolic and immune regulation, elucidating its molecular mechanisms and therapeutic implications in PCOS. By emphasizing the cross-system regulatory properties of the pathway, this work aims to inspire researchers to explore novel insights into PCOS pathogenesis and therapeutic targets.

## 2 Search methods

To ensure a comprehensive review of the literature, a systematic search was performed using the PubMed, Medline, and Embase databases for relevant articles published between 2010 and 2025. The search used a combination of keywords and Medical Subject Headings (MeSH) terms related to “polycystic ovary syndrome”, “infertility”, “ovarian follicular development”, “ovarian microenvironment”, “lipid metabolism”, “metabolic dysfunction-associated steatotic liver disease/MASLD”, “insulin resistance”, “hyperandrogenemia”, “adipose tissue”, “inflammation”, “macrophages”, and “Hippo signaling pathway” (including YAP/TAZ, MST1/2 and LATS1/2). The scope of the search encompassed original research (observational, epidemiological and experimental), reviews, systematic reviews, and meta-analyses. The inclusion criteria were limited to English-language articles presenting original data or seminal reviews, prioritizing those published in peer-reviewed journals with an emphasis on recent evidence. Additional information was obtained from references cited in the articles resulting from the literature search.

## 3 Results

### 3.1 Mechanism of Hippo signaling pathway transduction

**Figure 1:**
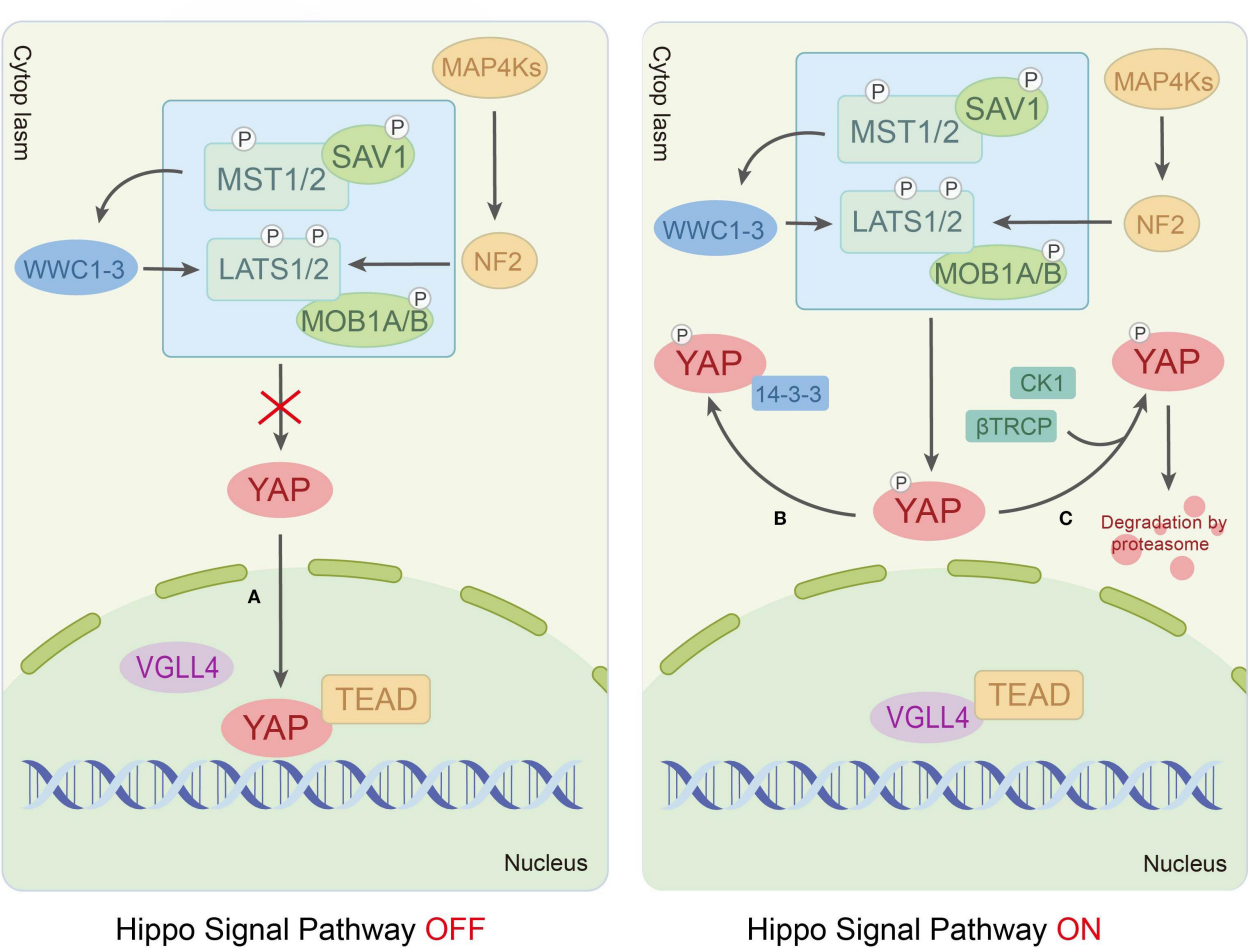
The Hippo signaling pathway transduction process.

In mammals, the Hippo signaling pathway is organized as a canonical serine/threonine kinase cascade. Within this cascade, the MST-LATS kinase can be activated by upstream signals, such as cell polarity, cell density, stress signals and mechanical cues (14, 15). Furthermore, the scaffolding protein Salvador homolog-1 (SAV1) binds to the mammalian Ste20-like kinase1/2 (MST1/2, orthologs of Hippo) via its SARAH domain, promoting MST1/2 autophosphorylation at Thr183/Thr180 sites to enhance kinase activity (16). Activated MST1/2 subsequently phosphorylates two critical regulatory domains of large tumor suppressor kinase 1/2 (LATS1/2, orthologs of Wts), including the Thr1079/Thr1041 sites in the hydrophobic motif (HM) and the Ser909/Ser872 sites in the activation loop (T-loop), thereby driving LATS1/2 activation (17). Additionally, MST1/2 phosphorylates the Thr12/Thr35 sites of the LATS1/2 coactivator Mps One Binder kinase activator-like 1A/1B (MOB 1A/B, orthologs of Mats), amplifying signaling through strengthened MOB1A/B-LATS1/2 interactions (18). Ultimately, the core effectors of the pathway, Yes-associated protein (YAP)/transcriptional co-activator with PDZ-binding motif (TAZ, also known as WWTR1), are phosphorylated by activated LATS1/2 at Ser127/Ser89 sites. This phosphorylation promotes YAP/TAZ cytoplasmic retention through 14-3–3 protein binding or facilitates their degradation via β-TrCP E3 ubiquitin ligase-mediated ubiquitination, suppressing their transcriptional activity (19). Conversely, Hippo pathway inactivation allows dephosphorylated YAP/TAZ to translocate into the nucleus, where they compete with VGLL4 (a Drosophila Tgi ortholog and transcriptional cofactor) to bind TEAD transcription factors (DNA-binding partners), regulating target genes governing cell proliferation, apoptosis and migration (10, 20) (**Figure 1**).

Recent studies have revealed the existence of more sophisticated regulatory mechanisms based on the classical Hippo signaling pathway. Qi et al. systematically analyzed the intermediate regulatory mechanism of the MST1/2-LATS1/2 kinase cascade, proposing two novel regulatory modules: HPO1 and HPO2. The HPO1 module involves WW and C2 domain-containing proteins (WWC1-3), which mediate LATS1/2-SAV1 interactions and localize the MST1/2-SAV1 complex to LATS1/2. The HOP2 module consists of neurofibromin 2 (NF2/Merlin) collaborating with mitogen-activated protein kinase 1-7 (MAP4K1-7, Hippo-like kinases), forming a redundant network with MST1/2 to regulate LATS1/2 activity (21–24). Furthermore, the transmembrane protein KIRREL1, a YAP/TAZ target protein, also enhances MST1/2-mediated LATS1/2 activation in a SAV1-dependent manner (25). Citron kinase (CIT), an AGC family kinase involved in mitotic regulation, exhibits dual regulatory roles in the pathway. On the one hand, it serves as an essential scaffolding protein bridging LATS2 and YAP during phosphorylation. On the other hand, it inhibits MST1-dependent LATS2 HM phosphorylation (26). This duality suggests that the effect of CIT on LATS2 may be dynamically modulated by the cellular microenvironment.

In conclusion, the Hippo signaling pathway governs the cellular localization and activity of the transcriptional coactivators YAP/TAZ via a highly conserved MST-LATS kinase cascade, which integrates diverse upstream signals to modulate the life activities of cells. Mounting evidence has further elucidated a sophisticated multi-tiered regulatory network, encompassing components such as the Hpo1 and Hpo2 modules, alongside factors including KIRREL1 and CIT. These discoveries substantially advance our comprehension of the pathway’s intricate complexity and context-dependent nature, while also offering novel insights into its molecular mechanisms in disease.

### 3.2 Hippo signaling pathway and ovarian dysfunction in PCOS

The pathogenesis of PCOS is rooted in its hallmark ovarian abnormalities, which provide a critical framework for investigating Hippo signaling dysregulation in this disorder.

#### 3.2.1 Hippo signaling in normal follicular development

During the reproductive cycle, key components of the Hippo signaling pathway are widely localized in ovarian cells, including oocytes, granulosa cells (GCs), theca cells and luteal cells, with dynamic expression patterns (27). For example, MST1 translocates to the nucleus from the cytoplasm gradually during oocyte development and achieves nuclear localization in the antral-stage oocytes (28). Similarly, YAP demonstrates stage-specific localization patterns in both oocytes and granulosa cells From primordial to preovulatory follicles, YAP progressively accumulates in the nucleus but relocates to the cytoplasm with markedly reduced expression in postovulatory luteal cells (28, 29). This observation is corroborated in a bovine ovary study, where microarray analysis revealed a significantly elevated YAP1 activation in larger developing follicles (5–10 mm) (30).

The Hippo signaling pathway governs the balance between primordial follicle dormancy and activation via mechanosensitive regulation. In mature ovaries, oocytes predominantly exist in a mechanically stressed state imposed by GCs and extracellular matrix (ECM), a condition critical for sustaining follicular dormancy and preserving female reproductive longevity (31). Subsequently, a series of studies confirms that mechanical signal during primordial follicle activation is involved in maintaining primordial follicle quiescence through activation of the Hippo signaling pathway (28, 32, 33). Furthermore, Liu et al. revealed that high cellular density promotes LATS1 SUMOylation at K830 residue (K829 in mice), enhancing kinase activity and amplifying Hippo signaling-mediated suppression of premature follicle activation (34–36).

Proliferation of GCs is also essential for primordial-to-primary follicle transition. Murine ovarian models demonstrate that activation of the Hippo signaling pathway downregulates pro-proliferative target genes *CCN2* and *CMYC*, thereby inhibiting GCs proliferation while promoting apoptosis (27, 37–39). Recent work by Chen et al. further demonstrates that YAP transcriptional activity prevents GCs apoptosis through NEDD8-mediated K159 neddylation (40). Thus, the Hippo signaling pathway participates in folliculogenesis by regulating GCs proliferation/apoptosis. Notably, YAP also serves as a crucial hub for follicle-stimulating hormone (FSH)- and luteinizing hormone (LH)-mediated follicular development. Physiologically, FSH suppresses YAP-TEAD transcriptional activity in GCs, upregulating steroidogenic enzymes (CYP11A1, HSD3B2 and CYP19A1) to enhance estradiol synthesis and dominant follicle selection (41, 42). Conversely, in cumulus cells FSH induces YAP-TEAD interactions, upregulating cumulus expansion genes (EGFR, ADAM17, EREG, and PTGS2) to promote oocyte maturation (43). Additionally, Hippo-YAP signaling also modulates LH secretion and function. In adenohypophysis, YAP/TAZ is a negative regulatory factor for LH secretion (44). During LH-induced ovulation, transient inactivation of the Hippo signaling pathway promotes nuclear YAP1 binding to the amphiregulin (Areg) promoter, thereby activating ERK1/2 signaling to induce LH target gene expression. Subsequent LH-induced cAMP/PKA signaling sequesters YAP in the cytoplasm, driving GC luteinization (45–47). Collectively, mature follicle formation and ovulation depend on precisely coordinating the activity of the Hippo signaling pathway (**Supplementary Table 1**).

#### 3.2.2 Ovulation dysfunction in PCOS

**Figure 2:**
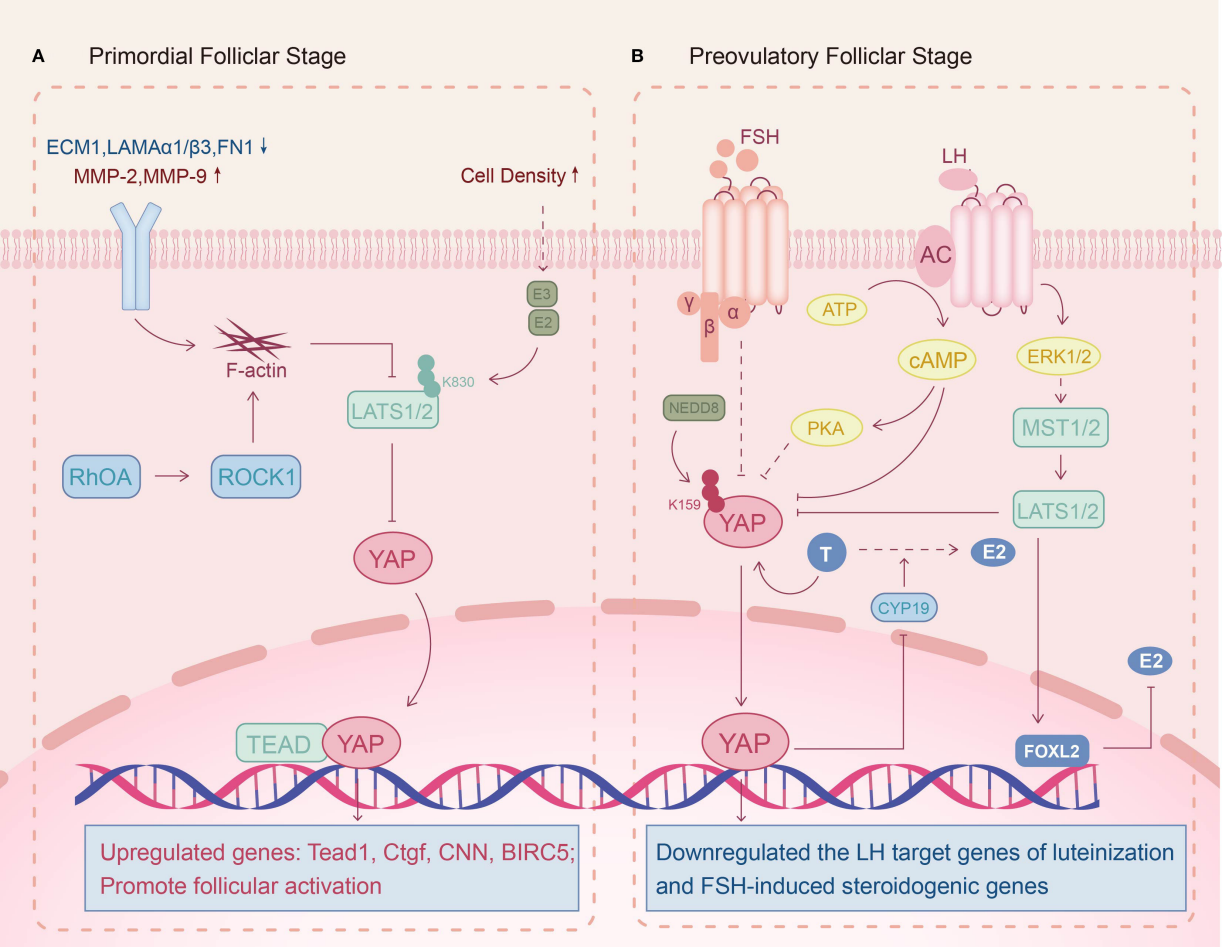
Potential molecular mechanisms of the Hippo signaling pathway in PCOS follicular development. **(A)** Primordial folliclar stage: Reduced extracellular matrix (ECM) stiffness maintains Hippo signaling activity and promotes follicular activation. **(B)** Preovulatory folliclar stage: Androgen-induced YAP upregulation inhibits FSH and LH effects on follicular development, impairing dominant follicle formation and ovulation.

Ovulatory dysfunction is a core diagnostic phenotype of PCOS, characterized by oligo-ovulation or anovulation, and its pathological mechanisms are closely associated with follicular developmental arrest and maturation impairment. Huang et al. observed that ovarian tissues of DHEA-induced PCOS murine models display an elevated YAP and phosphorylated YAP (p-YAP) expression but a significant reduction in p-YAP/YAP ratio (48). Mechanistically, this aberrant YAP1 activation in PCOS stimulates GCs hyperproliferation and downregulates luteinization-associated LH target genes (*CYP11A1, STAR, LHCGR, PGR*), which induces GC differentiation arrest, maintaining it in a non-luteinized state and triggering the pathological accumulation of immature ovarian follicles (49). Notably, impaired FSH signaling in PCOS synergistically contributes to dominant follicle selection failure (50). Recent studies reveal that YAP overexpression can impede follicular maturation by suppressing FSH responsiveness in GCs (42). In addition, a pathogenic androgen-YAP positive feedback loop may exist in PCOS. Jiang et al. found that androgens can attenuate YAP1 promoter methylation in a dose-dependent manner to increase YAP1 transcriptional activation in GCs (51). Concurrently, YAP-TEAD complex upregulation inhibits aromatase activity through suppressing CYP19A1 expression to affect the biotransformation of androgens to estrogens. This dual mechanism culminates in localized ovarian estrogen deficiency and androgen excess that obstruct follicle maturation and ovulation (42). Collectively, these findings suggest that abnormal YAP activation is intimately linked to ovulation dysfunction in PCOS (**Figure 2B**).

#### 3.2.3 Decreased ovarian reserve (DOR) in PCOS

Observational clinical cohort studies have revealed that some PCOS patients exhibit DOR, but the exact mechanism is unclear (52). Dysregulation of ECM homeostasis in GCs may contribute to this pathology. For PCOS, ECM-related genes are down-regulated in the GCs, including *ECM1*, laminin α3/β1 (*LAMA3/LAMB1*) and fibronectin 1 (*FN1*), while matrix metalloproteinases (*MMP-2, MMP-9*) are upregulated, indicating a reduction in ECM stiffness (53–55). Furthermore, genomic analysis identified that the Ras homology growth-related (*RHOG*) gene, which regulates actin cytoskeleton polymerization, is abnormally elevated in PCOS patients’ GCs (56). When GCs perceive a decreased mechanical stress, F-actin is induced to form and hinders the Hippo signaling pathway, thereby triggering premature activation of dormant follicles (7, 27, 57). Pharmacological induction of actin polymerization corroborates this mechanism, as enhanced YAP nuclear translocation drives follicular recruitment (58). Thus, Hippo pathway dysregulation in GCs likely contributes to the pathological early follicular recruitment observed in PCOS-associated DOR (**Figure 2A**).

#### 3.2.4 Ovarian microenvironmental perturbations in PCOS

The ovarian microenvironment provides nutritional support and signaling transduction essential for normal follicular development (59). Patil et al. found that follicular growth arrest, luteal insufficiency and recurrent miscarriage in PCOS are associated with impairment of the vascular system (60). Moreover, follicular fluid in PCOS displays a down-regulation of pro-angiogenic genes (*FGFR1, VEGFA, FN1*), reflecting compromised vascular development (60). Genome-wide association analysis further identified a significant association between vascular endothelial growth factor (*VEGF*) gene polymorphisms and PCOS susceptibility (61). Mechanistic investigations demonstrate that neovascularization is dependent on a feed-forward loop between YAP/TAZ and VEGF-VEGFR2 signaling in endothelial cells mediated by cytoskeletal dynamics, while YAP suppression disrupts angiogenesis (62, 63). Consequently, diminished pro-angiogenic factors in PCOS microenvironments may impede vascular sprouting by inhibiting YAP signaling in endothelial cells.

Emerging evidence implicates there are relationships between the ovarian microenvironment exposure to environmental contaminants, such as perfluoroalkyl and poly-fluoroalkyl substances (PFAS), zearalenone (ZEN), microplastics and phthalates, and the risk of PCOS, which involve the Hippo signaling pathway dysregulation (64–68). Firstly, Perfluorooctanoic acid (PFOA) exposure can result in ovarian fibrosis and DOR, which are associated with abnormally high expression of YAP (69, 70). Secondly, co-exposure of polystyrene nanoparticles (PS-NPs) and phthalates induced PCOS-like phenotypes in mice via ROS-Hippo signaling activation (66, 71). Finally, single-cell RNA sequencing identifies the Hippo signaling pathway disruption as the molecular basis for ZEN-induced primordial follicle assembly defects (72). These findings establish a theoretical framework linking environmental toxicants to PCOS through the modulation of the Hippo signaling pathway, advancing etiological and therapeutic insights.

Overall, the Hippo signaling pathway plays a central role in ovarian dysfunction associated with PCOS. Under physiological conditions, this pathway is instrumental in the precise regulation of follicular development, granulosa cell proliferation and differentiation, hormonal response, and the ovulation process. In PCOS, however, significant dysregulation of this pathway (e.g., abnormal nuclear localization and sustained activation of YAP) triggers a cascade of pathological alterations, including imbalances in granulosa cell proliferation and differentiation, premature follicular activation and impaired angiogenesis, etc. Collectively, these disruptions contribute to ovulatory dysfunction, DOR and an aberrant ovarian microenvironment. The above findings not only provide deeper insights into the pathogenesis of PCOS-related ovarian dysfunction but also establish a rational basis for developing novel therapeutic strategies aimed at the Hippo signaling pathway.

### 3.3 Hippo signaling pathway and metabolic dysregulation in PCOS

PCOS is not only a reproductive disorder, but also a systemic metabolic syndrome characterized by IR, hyperinsulinemia, dyslipidemia and obesity. Emerging evidence implicates the Hippo signaling pathway as a critical regulator of these metabolic perturbations (**Supplementary Table 2**).

#### 3.3.1 Lipid metabolism dysregulation in PCOS

**Figure 3:**
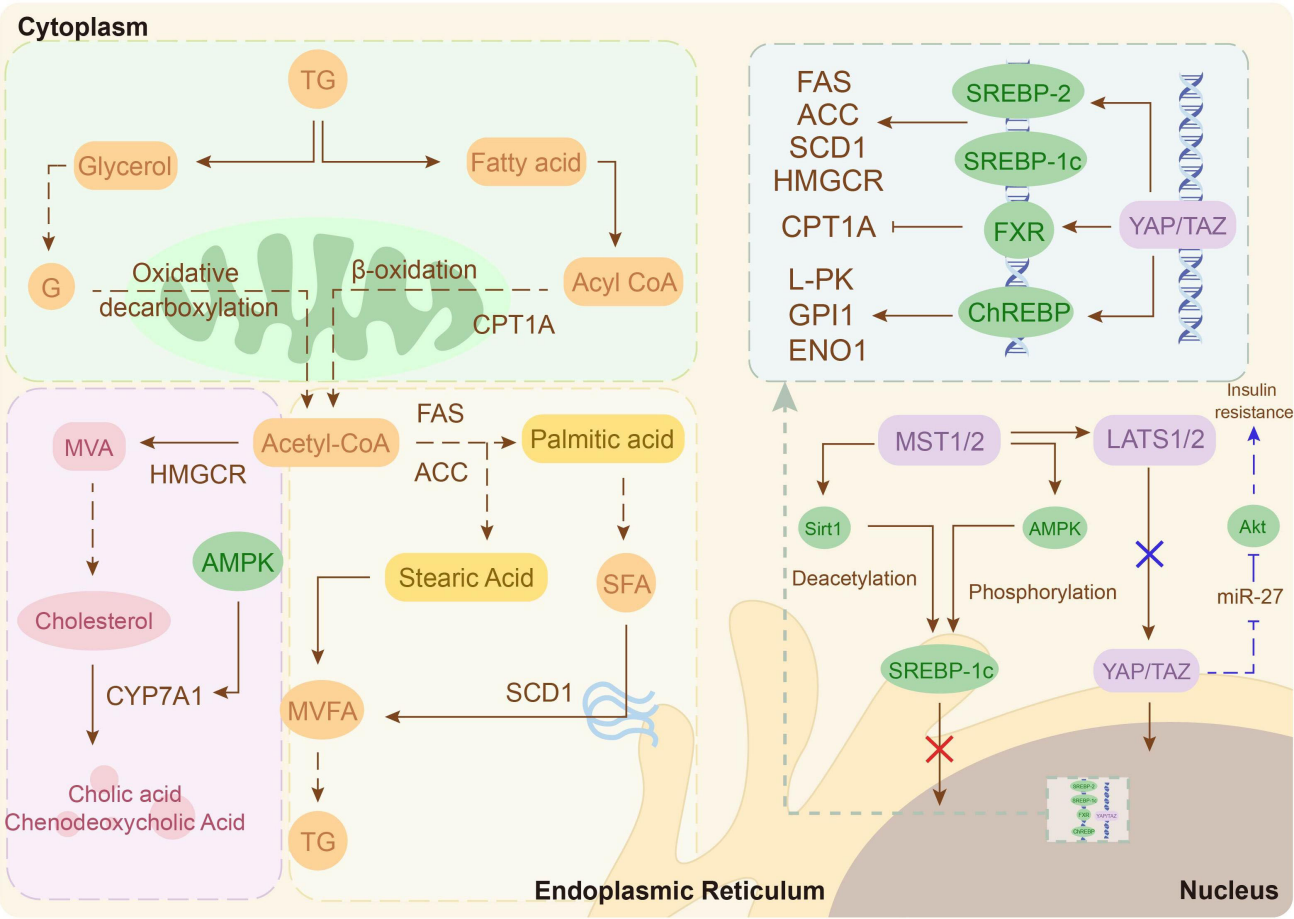
Potential molecular mechanisms of the Hippo signaling pathway in hepatic lipid metabolism. Hyperlipidemia inhibits Hippo signaling, activating YAP/TAZ and enhancing the transcriptional activity of SREBP-2, SREBP-1c and ChREBP, thereby driving hepatic lipid metabolism. Additionally, the Hippo signaling pathway inactivation suppresses insulin signaling, exacerbating insulin resistance (IR) (blue arrow).

The liver, a central organ in lipid homeostasis, exhibits Hippo-mediated dysregulation in PCOS (73). Clinical studies reveal a bidirectional association between PCOS and MASLD, though the underlying molecular mechanisms remain unclear (74, 75). Further exploration finds that hyperandrogenemia induces metabolic disruption in the liver of PCOS via increasing YAP expression and activity (73). Mechanistically, YAP is a co-activator of sterol regulatory element binding proteins (*SREBP-1c* and *SREBP-2*) and a nuclear cofactor for carbohydrate response element binding protein (*ChREBP*) that amplifies the expression of their target genes, thereby accelerating fatty acid and cholesterol production in hepatocytes (76, 77). In addition, inhibition of fatty acid oxidation mediated by the YAP-FXR axis can further exacerbate lipid deposition (78). In conclusion, the increased activity and expression levels of YAP in patients with PCOS are one of the major causes of their hepatic lipid metabolism disorders (**Figure 3**).

Obesity is another important phenotype of lipid disorders in PCOS, affecting approximately 50% of these patients (79). Its pathological changes are characterized by adipose tissue excessive expansion (hypertrophy and/or hyperplasia) (80). Despite the lack of studies on the Hippo signaling pathway in adipose tissue of obese PCOS patients, researchers have observed significantly elevated YAP activity in white adipose tissue of humans and mice with obesity (81). Mechanistically, high YAP activity can exacerbate adipocyte hypertrophy by inducing Wnt5a-1 expression (82, 83). Therefore, high YAP activity in adipocytes of obese PCOS patients may contribute to their fat accumulation.

**Figure 4:**
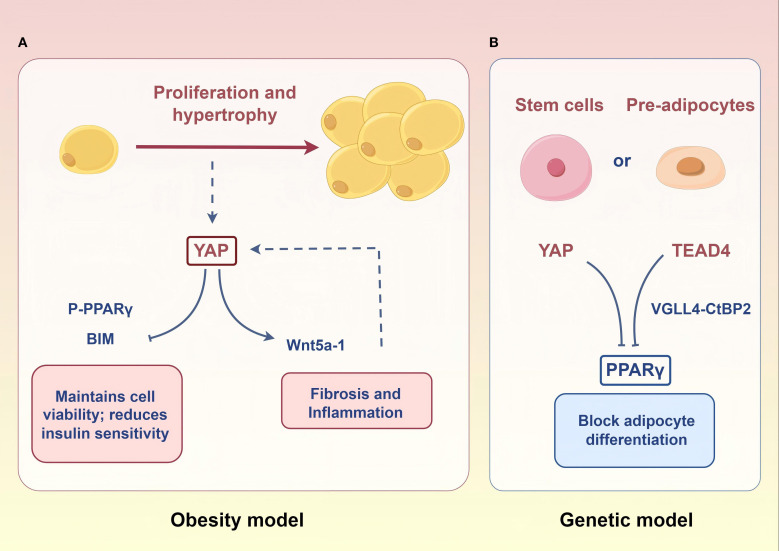
Potential molecular mechanisms of the Hippo signaling pathway in adipose tissue. **(A)** In the context of obesity, heightened YAP activity within mature adipocytes drives adipocyte hypertrophy and proliferation. **(B)** In a potential genetic model, elevated *PPARγ* expression in stem cells/pre-adipocytes of lean individuals enhances their differentiation into mature adipocytes. The downregulation of YAP and TEAD4 expression may potentiate this pro-differentiation effect. Figure was created by Figdraw (www.figdraw.com).

However, in normal-weight women with PCOS, adipogenic gene expression (*PPARγ, CEBPα, AGPAT2*) is abnormally elevated in their abdominal adipose-derived stem cells compared to healthy controls (84). Furthermore, a meta-analysis confirmed a significant association between *PPARG* gene polymorphisms and PCOS susceptibility (85). These imply that enhanced adipogenic tendency is a risk for non-obese patients. In recent years, a series of laboratory studies have demonstrated that the YAP-PPARγ regulatory axis is a key molecular mechanism for the differentiation of endocrine stem cells/pre-adipocytes to mature adipocytes (86–88). Therefore, there may be a lipogenic tendency mediated by the Hippo-YAP signaling pathway in preadipocytes of non-obese women with PCOS (**Figure 4**).

#### 3.3.2 Insulin resistance and hyperinsulinemia in PCOS

IR as a core phenotype of metabolic disorders in PCOS, involves functional dysregulation across multiple organs (89). Hepatic lipid accumulation promotes IR by inducing the Hippo signaling pathway inactivation to amplify miR-27-mediated suppression of Akt signaling (73). Moreover, insulin sensitivity and glucose homeostasis in adipose tissue are negatively correlated with YAP activity (90, 91). Skeletal muscle, as another major target organ for insulin, occurs IR is closely related to reduced YAP levels (92, 93). Mechanistically, inhibited YAP can lead to IR by inducing mitochondrial fatty acid oxidizing capacity dysfunction in skeletal muscle (93). Furthermore, although not clear in skeletal muscle, reduced YAP/TAZ activity has been shown to cause downregulation of IRS1 phosphorylation levels in endometrial cancer cells, which in turn affects insulin sensitivity (94). Notably, hepatic lipid accumulation, adipose tissue dysfunction, mitochondrial dysfunction and IRS1/PI3K/Akt signaling inhibition in skeletal muscle underlies IR pathogenesis in PCOS (91, 95, 96). Collectively, Hippo-YAP dysregulation underpins IR pathogenesis through hepatic steatosis, adipocyte dysfunction, and skeletal muscle metabolic inflexibility.

Hyperinsulinemia (HI), caused by pancreatic β-cell hypersecretion, exhibits a bidirectional pathological association with IR (89). Emerging clinical evidence suggests HI may precede IR onset in PCOS, correlating with hyperandrogenemia (97, 98). Prenatal androgen exposure induces β-cell apoptosis, whereas postnatal exposure triggers compensatory β-cell hyperplasia and HI (99–101). Mechanistic studies demonstrate that the Hippo pathway collaborates with neurogenin 3 (*NGN3*) to silence YAP during endocrine lineage specification, which is a prerequisite for functional β-cell differentiation (102–104). Conversely, aberrant YAP activation disrupts its functional maturation (105). Intriguingly, the upregulation of YAP expression in mature β-cells stimulates proliferation without affecting function (106–108). Given androgen-induced YAP activation in hepatic and ovarian tissues, we propose that hyperandrogenemia drives HI via ectopic YAP activation in pancreatic β-cells, establishing a self-reinforcing endocrine-metabolic loop.

In summary, dysregulation of the Hippo signaling pathway appears to be a critical node in the molecular mechanisms underlying metabolic disturbances in PCOS. This pathway not only contributes to hepatic lipid accumulation by regulating lipid synthesis and lipolysis, but also mediates adipose tissue dysfunction. Furthermore, it extensively influences insulin signaling transduction in peripheral tissues, promoting the development of systemic insulin resistance and hyperinsulinemia. These mechanisms highlight the significant role of the Hippo signaling pathway in the systemic metabolic dysregulation of PCOS, providing new perspectives on the disorder’s molecular underpinnings.

### 3.4 Hippo signaling pathway and chronic low-grade inflammation in PCOS

**Figure 5:**
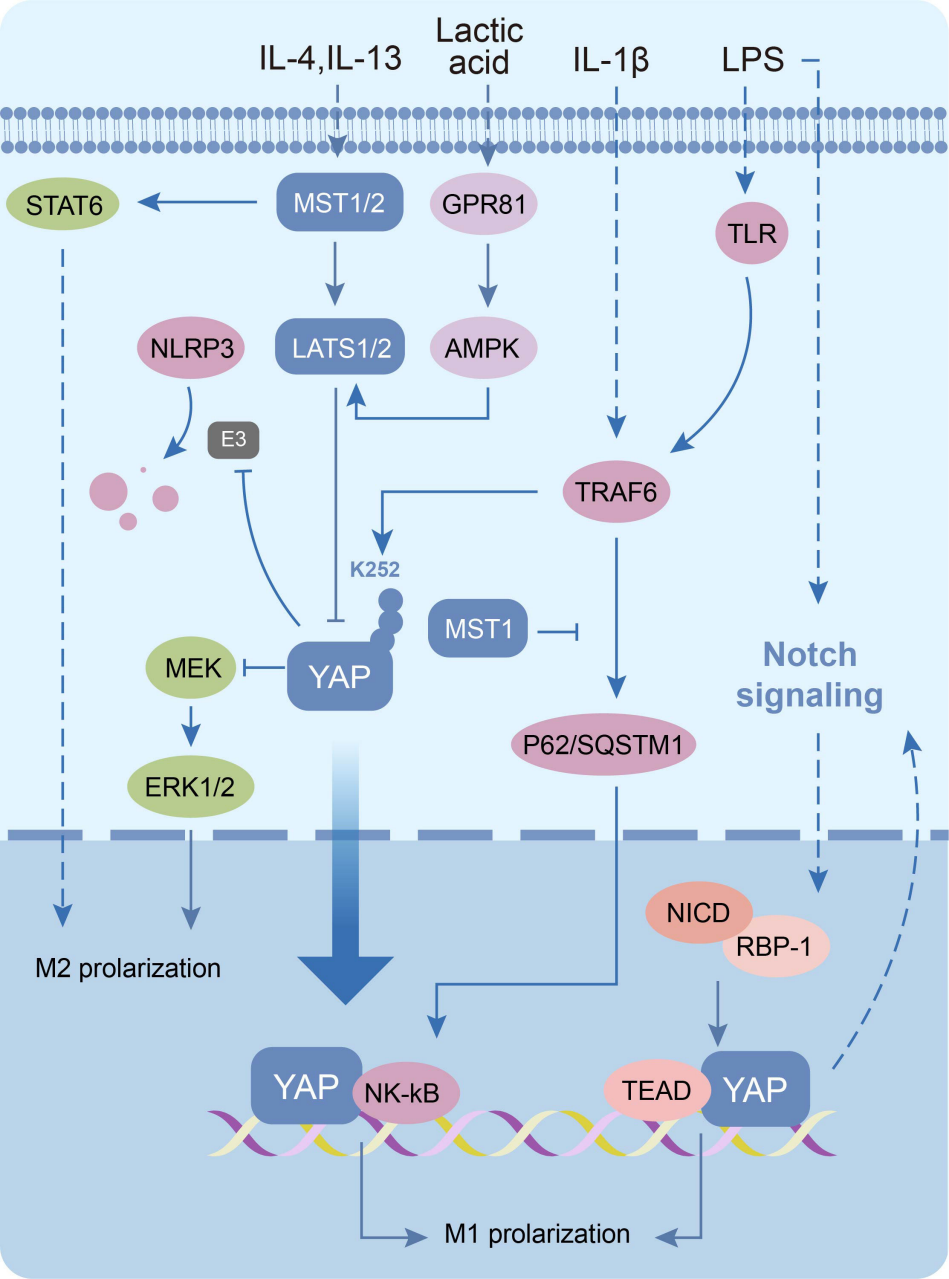
Molecular mechanisms of the Hippo signaling pathway in macrophages. The Hippo signaling pathway inactivation induces macrophage M1 polarization as well as increased levels of inflammatory vesicle NLRP3. Conversely, the pathway activation promotes macrophage M2 polarization.

In recent years, researchers have identified chronic low-grade inflammation as one of the central aspects of PCOS pathophysiology, forming a complex network with IR, hyperandrogenemia and metabolic abnormalities (109). Chronic low-grade inflammation in PCOS is mainly manifested in the form of increased levels of various inflammatory factors (eg: IL-6, IL-1β, IL-18) and dysregulated M1/M2 macrophage polarization in ovarian microenvironments (110, 111). Mechanistic studies reveal YAP drives inflammation through dual mechanisms. During LPS/TFN-γ-induced pro-inflammatory M1 polarization, YAP/TAZ overexpression stabilizes cytosolic NLRP3 inflammasomes by inhibiting β-TrCP1-mediated ubiquitination in the cytoplasm; as well as combining with TEAD to directly activate *IL-6* transcription via promoter binding in the nuclear (112, 113). Furthermore, YAP amplifies inflammation through NF-κB and Notch1 pathway activation (114–116). Notably, the inflammatory factor IL-1β can in turn promote macrophage M1 polarization by inducing ubiquitination of YAP at the K252 site to increase activity (117). Conversely, IL-4/IL-13-induced anti-inflammatory M2 polarization requires deregulating YAP inhibition of the MEK/ERK pathways, thereby restoring anti-inflammatory gene expression (*Arg, Egr2, Cd206, Ym1, Fizz1*) (118, 119). These findings implicate that YAP-mediated macrophage polarization imbalance may be a pivotal mechanism sustaining chronic inflammation in PCOS (**Figure 5**) (**Supplementary Table 3**).

### 3.5 Therapeutic prospects in PCOS: targeting the Hippo-YAP signaling pathway

#### 3.5.1 Modulating follicular development to restore fertility

Follicular development modulation represents a cornerstone for addressing PCOS-related infertility. Laparoscopic ovarian drilling (LOD), as a surgical intervention for clomiphene citrate (CC)-resistant patients, inhibits Hippo signaling to promote primordial follicle activation, though its efficacy remains debated (7, 120–123). Emerging evidence suggests that the Hippo signaling pathway activation represents an effective therapeutic mechanism in PCOS patients with impaired follicular development. Huang et al. observe that verteporfin (a YAP-TEAD interaction inhibitor) treatment in PCOS mice can reduce serum anti-Müllerian hormone (AMH) levels and restore follicular growth (48). Furthermore, YAP also serves as a molecular hub of biopharmaceutical and nutraceutical interventions for PCOS. Firstly, anti-growth factor-releasing peptide antibodies alleviate PCOS phenotypes in murine models, such as weight gain, estrous cycle disruption, ovarian morphologic aberrations and hormonal imbalances, via silencing Gαq/11 or YAP in GCs (124). Secondly, adding n-3 polyunsaturated fatty acids (PUFAs) to the diet significantly alleviated hormonal and estrous cycle disturbances in PCOS mice by reducing YAP1/Nrf2 signaling in GCs (125). Therefore, targeting the Hippo-YAP signaling pathway is a promising therapeutic strategy for restoring fertility in PCOS patients.

#### 3.5.2 Metabolic correction: adipose and insulin signaling

Weight management constitutes the cornerstone of metabolic intervention in PCOS, particularly in patients with comorbid overweight/obesity (79). Current therapies reduce adipocyte hypertrophy but fail to modulate hyperplasia (80, 126). A mechanistic study by Wang et al. has revealed that YAP inhibition selectively induces apoptosis in mature adipocytes (81). Notably, the adipogenic marker PPARγ in preadipocytes exhibits a significant correlation with PCOS metabolic parameters, which is negatively regulated by YAP and TEAD4 (127–129). In addition, MST1/2 gene deletion enhances adipocyte mitochondrial autophagy activity through a non-YAP-dependent pathway, thereby elevating the efficiency of energy metabolism and inhibiting dietary obesity (130). Liraglutide as a common insulin sensitizer in PCOS patients, despite its known glucose-lowering effect, also inhibits the proliferation of preadipocytes through activation of the Hippo signaling pathway but also accelerates adipogenic differentiation (131).

IR management is the center of PCOS metabolic intervention. Experimental evidence indicates that adipocyte-specific YAP/TAZ knockout significantly enhances insulin sensitivity in obese murine models (90, 132). In addition, both clinical observations and animal studies demonstrate reduced proportions of insulin-sensitive type I muscle fibers in PCOS, which may be an important pathological basis for skeletal muscle IR (96, 133). Remarkably, LATS1/2 knockout in mice can significantly increase the percentage of type I muscle fibers in skeletal muscle (134). Thus, precise modulation targeting the Hippo signaling pathway may provide an innovative therapeutic strategy to ameliorate PCOS metabolic disorders.

#### 3.5.3 Anti-inflammatory interventions

Although current clinical guidelines for PCOS have not yet incorporated systemic anti-inflammatory treatment regimens, accumulating experimental evidence underscores the pivotal role of inflammatory modulation in improving reproductive and metabolic outcomes in PCOS (109, 126). Recently, Wang et al. proposed a potential molecular pathway of PUFAs in PCOS treatment. Their findings demonstrate that PUFAs significantly upregulate anti-inflammatory gene expression and induce macrophage M2-like polarization by inhibiting RhoA-YAP1 signaling (135, 136). This implies a potential therapeutic value of the Hippo-YAP signaling pathway remodeling immunocyte phenotype for anti-inflammatory interventions in PCOS.

#### 3.5.4 Comorbidity management

PCOS combined with MASLD significantly constrains therapeutic options, exacerbates disease management complexity and amplifies long-term adverse outcomes (137). Researchers have found that hyperlipidemia and IR are shared pathological foundations between PCOS and MASLD, suggesting potential synergistic therapeutic targets (138). In clinical practice, glucagon-like peptide-1 (GLP-1) receptor agonists, thiazolidinediones, and statins have demonstrated dual therapeutic effects, their side-effect profiles necessitate novel approaches (139). Animal experiments have confirmed that YAP knockout in MASLD model mice can effectively reduce hepatic triglyceride (TG) and Perilipin 2 (PLIN2) levels and ameliorate lipid metabolism disorders (140). Moreover, emerging therapeutic approaches utilizing chrysanthemum lactone (PAR), anti-miR-199a-5p exosome and Hep@PGEA/MST1 nanocarrier demonstrate reducing hepatic lipid burden effects by disrupting the Hippo signaling pathway (141–143). These findings collectively propose that targeting the expression of Hippo signaling components to regulate metabolic disorders in the liver may be a novel way to treat PCOS-MASLD comorbidities.

Notably, LATS1 activity in GCs is negatively correlated with steroidogenic acute regulatory protein (StAR)-mediated estrogen synthesis (144). However, pathological estrogen elevation significantly increases the risk of ovarian hyperstimulation syndrome (OHSS), a serious complication frequently observed in PCOS patients undergoing *in vitro* fertilization (IVF)/intracytoplasmic sperm injection (ICSI) treatments (145). Consequently, activation of LATS1 in GCs may be a preventive strategy against OHSS during PCOS patients’ assisted reproductive therapy.

In conclusion, targeting the Hippo-YAP signaling pathway represents a promising multifaceted therapeutic strategy for PCOS, encompassing key areas such as reproductive function, metabolic regulation, inflammatory response and comorbidity management. Specifically, modulation of this pathway could potentially promote follicular development, improve metabolic disorders, regulate immune responses and prevent complications such as OHSS and MASLD.

The current clinical management of PCOS is still based on symptomatic relief as the main goal with limited therapeutic options (146). To address this current situation, developing stage-specific and tissue-targeted Hippo pathway modulation strategies holds significant clinical promise. However, the clinical translation of such strategies faces several challenges, including limitations in targeting precision, delivery efficiency, biocompatibility, safety, stability, and scalable production. Therefore, future research should emphasize multidisciplinary collaboration to optimize delivery systems, thereby providing more robust and precise tools for disease treatment. Furthermore, significantly upregulated YAP expression has been observed in ovarian granulosa cells of PCOS patients, suggesting that quantitative assessment of Hippo signaling biomarkers (e.g., p-YAP and YAP levels) may facilitate early diagnosis and real-time therapeutic monitoring (51).

## 4 Conclusions

This review systematically delineates the multidimensional regulatory mechanisms of the Hippo signaling pathway and its core components (MST1/2, LATS1/2, YAP/TAZ) in the pathogenesis of PCOS. In reproductive dysfunction, Hippo dysregulation drives primordial follicle depletion, granulosa cell apoptosis-proliferation imbalance, and anovulation, while also mediating environmental toxicant-induced ovarian injury. Metabolically, this pathway is involved in systemic metabolic disturbances in PCOS by regulating pancreatic β-cell function, adipose tissue differentiation/function, skeletal muscle insulin sensitivity, and hepatic lipid metabolism. Immunologically, YAP-driven M1 macrophage polarization might emerge as a pivotal mechanism underlying chronic low-grade inflammation in PCOS. Collectively, Hippo signaling emerges as a molecular linchpin integrating the reproductive-metabolic-immune axis in PCOS, establishing a novel therapeutic paradigm for targeted interventions (**Table 1**).

**Table 1:** Effects of core components of the Hippo signaling pathway on follicular development.

Study (first author, year)	Model system	Gene expression	Findings
Cai, Jun-Hong et al. (2022) (144)	KGN and SVOG cell line	LATS1 ↑	Suppressed the secretion of estrogen in GCs
Ai A,2018 (37)	Primary mouse ovarian granulosa cells	LATS1↓	Promoted GCs proliferation
Ren P,2024	Primary hen ovarian granulosa cells	LATS2↓	Promoted GCs autophagy and apoptosis
Bao D,2023 (38)	Primary human ovarian granulosa cells and KGN cell line	SAV↓	Promoted GCs proliferation; inhibited GCs apoptosis
Hu LL (2019) (27)	Mouse model—*in vitro* ovary culture	YAP1↑	Increased primary follicles; decreased primordial follicles
		YAP1↓	Increased primordial follicles; decreased primary follicles
Devos M (2023) (39)	Mouse model—*in vitro* ovary culture	YAP1-TEAD↓	Decreased expression Ccn2, Cmyc
Sun T (2019) (45)	Mouse model—*in vitro* cumulus-oocyte complexes and mural cell culture	YAP1-TEAD↓	Inhibited GCs proliferation; triggered cumulus cells premature differentiation
Lv X (2019) (29)	Mouse model—*in vitro* ovary culture	YAP1↓	Disrupted ovarian follicle development
	KGN cell line and Primary mouse ovarian granulosa cells	YAP1↑	Promotes proliferation but suppresses differentiation of granulosa cells
de Andrade LG (2022) (41)	Primary bovine ovarian granulosa cells	YAP1-TEAD↓	Decreased mRNA expression CTGF, CYR61and ANKRD1, but increased CYP19A1
Mizutani T (2023) (42)	KGN cell line	YAP/TAZ↓ or TEAD↓	Increased mRNA expression CYP19A1, CYP11A1 and HSD3B2 (steroidogenic enzyme-encoding genes); increased 17β-estradiol production
Koch J (2022) (43)	Primary bovine cumulus-oocyte complexes (COCs)	YAP1-TEAD↓	Decreased the expression of critical FSH-induced cumulus expansion–related genes (EGFR, ADAM17, EREG, PTGS2, HAS2, PTX3 and PLAT)
Dos Santos EC (2022) (46)	Bovine model	YAP1-TEAD↓	Decreased ovulation in cattle
	Primary bovine ovarian granulosa cells		Inhibited the expression of classic LH-induced preovulatory genes (EREG and PTGS2)
Godin P (2022) (47)	Yap1^f/f^;Taz^f/f^ mouse modal isolate granulosa cells	YAP↓	Decreased protein expression Areg, Pgr, Ptgs2 and Lhcgr; blunt LH responsiveness
Plewes MR (2019) (30)	Primary bovine ovarian granulosa cells	YAP1/TAZ↓	Inhibited TGFα-induced GCs proliferation and FSH-induced estradiol production
Chen, Mengjuan et al. (2024) (40)	KGN cell line	YAP↓	Promoted GCs apoptosis
Cheng, Yuan et al. (2015) (58)	Constitutive active (CA)-YAP mouse modal	YAP1↑	Enhanced follicle development
Hu, Liao-Liao et al. (2019) (27)	Mouse model—*in vitro* ovary	YAP↓	Suppressed primordial follicle activation

The symbols provided in the table “↑” are overexpression and “↓” are suppression of expression.

## Glossary

PCOS: Polycystic ovary syndrome
IR: Insulin Resistance
GWAS: Genome-wide association study
SAV1: Salvador homolog-1
MST1/2: Mammalian Ste20-like kinase1/2
LATS1/2: Large tumor suppressor kinase 1/2
HM: Hydrophobic motif
T-loop: The activation loop
MOB 1A/B: Mps One Binder kinase activator-like 1A/1B
YAP: Yes-associated protein
TAZ: Transcriptional co-activator with PDZ-binding motif
WWC1-3: WW and C2 domain-containing proteins
NF2/Merlin: Neurofibromin 2
MAP4K1-7: Mitogen-activated protein kinase 1-7
CIT: Citron kinase
GCs: Granulosa cells
ECM: Extracellular matrix
FSH: Follicle-stimulating hormone
LH: Luteinizing hormone
Areg: Amphiregulin
p-YAP: Phosphorylated YAP
LAMA3/LAMB1: Laminin α3/β1
FN1: Fibronectin 1
MMP: Matrix metalloproteinases
RHOG: Ras homology growth-related
VEGF: Vascular endothelial growth factor
PFAS: Perfluoroalkyl and poly-fluoroalkyl substances
ZEN: Zearalenone
PFOA: Perfluorooctanoic acid
PS-NPs: Polystyrene nanoparticles
MASLD: Metabolic dysfunction-associated steatotic liver disease
SREBP-1C/2: Sterol regulatory element binding protein 1C/2
ChREBP: Carbohydrate response element binding protein
HI: Hyperinsulinemia
NGN3: Neurogenin 3
LOD: Laparoscopic ovarian drilling
CC: Clomiphene citrate
AMH: Anti-Müllerian hormone
PUFAs: Polyunsaturated fatty acids
GLP-1: Glucagon-like peptide-1
TG: Triglyceride
PLIN2: Perilipin 2
PAR: Chrysanthemum lactone
StAR: Steroidogenic acute regulatory protein
OHSS: Ovarian hyperstimulation syndrome
IVF: In vitro fertilization
ICSI: Intracytoplasmic sperm injection
